# Studies in Pathological Anatomy

**Published:** 1878-07

**Authors:** 


*Studies in Pathological Anatomy.* By FRANCIS DELAFIELD, D. M. March, April and May, 1878, No. 2, 3, and 4. New York: Wm. Wood & Co. Subscription $5,00 a year. Single number 50 cents.

We have already expressed our favorable opinion of this excellent series, and can see no reason to change, indeed, each number increases the esteem in which we hold it. The plates are all well executed, truthful and instructive, while the text fully explains the subjects portrayed.

No. 2 contains five plates from IV to VIII, giving the anatomy of the pleura and shows the endothelium, connective tissue cells, and lymphatics of this important structure.

No. 3 illustrates the changes in the plura due to inflammatory action. Plate IX shows the new cells from Pleurisy in the Dog, 24 hours. Plate X Layer of cells and fibrine from Pleurisy in the Dog 3d day. Plate XI gives an idea of the formation of a layer of new tissue as seen in Pleurisy 5th day; Plate XII indicates the nature of endothelial and connective cells as found in pleurisy of the Dog, 7, 9 days.

No. 4 illustrates the radical changes due to empyema as seen in a dog after 24 hours, and again at ten days, and in Plate XV a vertical section of human pleura changed by empyema.

These numbers more than meet the expectations raised by the first, and will remain a worthy monument to the industry of the talented author.

